# In-Country Method Validation of a Paper-Based, Smartphone-Assisted Iron Sensor for Corn Flour Fortification Programs

**DOI:** 10.3390/foods11030276

**Published:** 2022-01-20

**Authors:** Anna W. Waller, Marcela Gaytán-Martínez, Juan E. Andrade Laborde

**Affiliations:** 1 Department of Food Science and Human Nutrition, University of Illinois at Urbana-Champaign, Urbana, IL 61801, USA; awaller2@illinois.edu; 2 Posgrado en Ciencia y Tecnología de los Alimentos, Research and Graduate Studies in Food Science, School of Chemistry, Universidad Autónoma de Querétaro, Querétaro 76010, Mexico; marcelagaytanm@yahoo.com.mx; 3 Food Science and Human Nutrition Department, University of Florida, Gainesville, FL 32611, USA

**Keywords:** paper-based assay, smartphone, iron, fortification, validation, colorimetric assay, corn flour

## Abstract

Food fortification in low-income settings is limited due to the lack of simple quality control sensing tools. In this study, we field validated a paper-based, smartphone-assisted colorimetric assay (Nu3Px) for the determination of iron in fortified flours against the gold standard method, atomic emission spectrometry (AES). Samples from commercial brands (*n* = 6) were collected from supermarkets, convenience stores, and directly from companies in Mexico and characterized using both Nu3Px and AES. Nu3Px’s final error parameters were quantified (*n* = 45) via method validation final experiments (replication and comparison of methods experiment). Qualitative pilot testing was conducted, assessing Nu3Px’s accept/reject batch decision making (accept ≥ 40 μg Fe/g flour; reject < 40 μg Fe/g flour) against Mexico’s fortification policy. A modified user-centered design process was followed to develop and evaluate an alternative sampling procedure using affordable tools. Variation of iron content in Mexican corn flours ranged from 23% to 39%. Nu3Px’s random error was 12%, and its bias was 1.79 ± 9.99 μg Fe/g flour. Nu3Px had a true mean difference from AES equal to 0 and similar variances. AES and Nu3Px made similar classifications based on Mexico’s policy. Using simple, affordable tools for sampling resulted in similar output to the traditional sampling preparation (r = 0.952, *p* = 0.01). The affordable sample preparation kit has similar precision to using analytical tools. The sample preparation kit coupled with the smartphone app and paper-based assay measure iron within the performance parameters required for the application to corn flour fortification programs, such as in the case of Mexico.

## 1. Introduction

Micronutrient deficiencies continue to afflict populations living in low- and middle-income countries [1]. Though other strategies to address it exist, food fortification has been heralded as the most cost-effective strategy to improve micronutrient status among vulnerable populations [2]. Despite its effectiveness, the food industry, as well as local governments, lack tools to monitor fortified food entering the different markets [3]. This is partially due to the limited availability of affordable and valid analytical and sensing tools to support these efforts [4,5].

Sensors are tools frequently used in the food and agriculture industry in recent decades for monitoring several important factors such as environmental changes (e.g., temperature and moisture), quality (e.g., texture and nutrient content), and safety (e.g., microbial and chemical hazards) of food products. The advantages to using sensors are that they are often low cost and provide fast, actionable data [6,7]. Sensor development includes the establishment of several performance parameters such as accuracy, reliability, specificity, linearity, dynamic range, and sensitivity [5]. In the case of low-income settings, the World Health Organization (WHO) has provided guidance under the ASSURED criteria for the development of diagnostic technologies (Affordable, Sensitive, Specific, User friendly, Rapid and Robust, Equipment-free, and Deliverable to End Users). These criteria help guide developers in the design of more impactful sensing tools, especially within limited resource settings [8].

Due to their ease of use and accessibility, smartphone-based sensors have garnered much interest in recent decades. These sensors can be categorized as electromagnetic, audio frequency, optical, or electrochemical [9]. In all cases, the smartphone acts as the sensor’s detection instrument, providing a data collection system that is familiar to the end user [10]. In 2014, it was estimated that 1.85 billion people used a smartphone [11]. Unarguably, some of the known advantages of smartphone-based sensors include their low cost and wide access [12]. Minimal training is required for using a smartphone as the sensor’s detection instrument, which reduces implementation costs [10]. Therefore, smartphone-based sensors fulfill WHO’s ASSURED criteria, strengthening their feasibility and applicability in resource-limited settings. Recent paper-based assays designed for food matrices have focused on the detection and quantification of additives (i.e., food colorings), pathogens, pesticides, herbicides, and toxic trace metals in foods [13,14,15,16]. Due to the lack of commercially available ASSURED-designed sensors to detect nutrients in fortified foods, our team developed a paper-based, smartphone-assisted assay for the determination of iron in fortified foods (also known as Nu3Px) [17]. The chemicals used in the ferrozine reaction were embedded in the paper sensor to measure different iron formulations employed in food fortification. The reaction started by placing a micro-aliquot of acidified samples on the paper. A few seconds later, contents turned magenta, and this color change was linearly associated with the concentration of iron present in the sample. With the help of an app, the smartphone was used to take a digital photo of the colored areas, which then were further processed using a color: iron concentration algorithm into a final iron concentration in the sample. This user-centered design followed and expanded WHO’s criteria as a validation step was included [5,17].

There are several design challenges to overcome in terms of the output’s reliability and accuracy of smart-based sensors. Reigning lighting conditions as well as the type of phone camera and flash, for example, can influence the photo rendition, which instead can affect processing and final result [12]. As this is a known factor, different teams have designed simple boxes that accommodate the sensor height and lighting to obtain the most reliable exposure [18,19]. Alternatively, other teams have resorted to using image scanners instead of a smartphone camera to work around this issue [20,21]. Beyond smartphone-related challenges, another hurdle in the development of ASSURED-designed sensors is the sample preparation, especially when measuring tools such as analytical balances and pipettes are not available. Altogether these challenges unduly influence the validity of measurements and thus the likelihood of commercialization [22,23].

Method validation consists of a series of experiments to prove that a new analytical method can provide accurate, reliable, and specific results for its intended application within allowable error limits. The error is then quantified by calculating both random and systematic error, which sum to the total error of a new method. These experiments include but are not limited to testing accuracy, precision, specificity, limit of detection, limit of quantification, linearity and range, ruggedness, and robustness. Best practices of new method adoption include the determination of these performance parameters [24].

The Nu3Px assay and smartphone app have been evaluated in terms of their preliminary performance parameters (i.e., specificity, linearity, dynamic range, sensitivity, reliability, and accuracy) [17]. These findings warrant further exploration of the final steps of method validation, including systematic error (SyE) and random error (RE) quantification, as described by Westgard [24], using a large number of actual field samples. The final error quantification experiments are comprised of the comparison of methods experiment and between-day replication experiment. The studies presented here were aimed at validating the Nu3Px sensor, in which we included and validated a sample collection tool kit that is aligned with WHO’s ASSURED criteria and used commercial corn fortified samples from Mexico. Ultimately, this technology can support monitoring and evaluation efforts of food fortification programs worldwide.

## 2. Materials and Methods

### 2.1. Collection and Characterization of Mexican Corn Flour Samples

Samples (*n* = 25) of corn flour from six commercial brands were collected from supermarkets, convenience stores, and directly from the companies in the Querétaro region, Saltillo, and Cuetzalan, Mexico. Convenience sampling was used to collect samples. Using atomic emission spectroscopy (AES, AOAC Official Method 984.27 [25]), the following mineral concentrations were measured: nitrogen, phosphorus, magnesium, potassium, calcium, sulfur, boron, manganese, copper, zinc, aluminum, sodium, and iron. Briefly, samples (0.3 g) were wet-ashed using 5 mL trace mineral-grade nitric acid and 2 mL 30% hydrogen peroxide. After closed vessel ashing, samples were diluted with ultrapure water to fit external standard curves for selected elements (Sigma-Aldrich, St. Louis, MO, USA). Samples were injected into an atomic emission spectrometer 4100 MP-AES (Agilent Technologies, Santa Clara, CA, USA) equipped with a plasma torch, a standard glass concentric nebulizer, and a cyclonic spray chamber. The MP-AES was calibrated using a diluted ICP-OES wavelength calibration solution (1:10 *v*/*v*) as an internal standard. Multielement standards were purchased from Sigma-Aldrich (St. Louis, MO, USA) to create external calibration curves. Nitrogen was measured in a CE440 Elemental Analyzer (Exeter Analytical, Inc., Chelmsford, MA, USA) based on thermal conductivity detection after combustion and reduction. All analyses used double deionized water. All glassware was either washed with acid solution prior to use or exchanged for plastic counterparts.

### 2.2. Replication Experiment (Determination of RE)

Using characterized samples, the Nu3Px sensor’s measurements were evaluated against AES by running tests to show between-day variability (coefficient of variation, CV_b_) based on 3 replicates collected at different times over 2 weeks of the same sample [24]. While the within-day CV% (CV_w_%) served as a preliminary measure of precision [17], CV_b_ (%) serves as the final indicator of random error as is customary in validation experiments [24].

### 2.3. Comparison of Methods Experiment (Determination of SyE)

The systematic error was determined by a comparison of methods, as described by Westgard [24]. The constant and proportional error for iron determination using the paper-based assay was assessed by using in-country corn samples from various companies and collection time points. A plot was constructed with the AES measurement on the x-axis and the paper-based measurement on the y-axis. A linear regression line was fit, where the slope indicates a proportional error (deviation from 1) and the y-intercept indicates constant error (deviation from 0), whereby proportional error and constant error together sum to SyE. However, if the linear regression coefficient (R^2^) is less than 0.99, a better indication of bias is by determining the mean and standard deviation of the differences between measurements from both methods. Then the total analytical error (TE) was determined by totaling CV_b_ (RE, replication experiment) and bias (SyE, comparison of methods experiment) [24].

Results from the comparison of methods experiment were used to make decisions whether a food processor (i.e., the technician who fortifies the flour) would hypothetically reject or accept a batch of corn flour based on Mexico’s current policy, in which batches fortified under 40 μg Fe/g flour would be rejected [26]. Hypothetical decisions were made using both AES and Nu3Px analyses. For each sample, results from AES analysis were considered the “true” result. If the Nu3Px analysis agreed with AES, it was considered a “true” accept or reject. If the Nu3Px analysis resulted in a different decision from AES, it was considered a “false” accept or reject. From these pass/reject matches or non-matches between tools, several qualitative performance parameters were assessed, including false-positive rate, false-negative rate, sensitivity, and specificity [27].

False-positive rate or α, a type I error, [28] refers to the probability that a batch is rejected by Nu3Px, but that has been accepted by AES (Equation (1)):

(1) $$\mathrm{False}\text{-}\mathrm{positive}\;\mathrm{rate},\;\alpha =\frac{\mathrm{fp}}{\mathrm{tn}+\mathrm{fp}},$$

in which fp refers to the number of false-positive test samples and tn refers to the true known number of negative test samples.

False-negative rate, also known as β, or a type II error, ref. [28] refers to the probability that a batch is accepted by Nu3Px, but that has been rejected by AES (Equation (2)):

(2) $$\mathrm{False}\text{-}\mathrm{negative}\;\mathrm{rate},\;\beta =\frac{\mathrm{fn}}{\mathrm{tp}+\mathrm{fn}},$$

in which fn refers to the number of false-negative samples and tp refers to the true known number of positive samples.

Sensitivity, or an assay’s power, [28] refers to the probability that a batch is accepted by Nu3Px and also by AES (Equation (3)):

(3) $$\mathrm{Sensitivity}=\frac{\mathrm{tp}}{\mathrm{tp}+\mathrm{fn}}=1-\beta ,$$

Finally, specificity refers to the probability that a batch is rejected by Nu3Px and also by AES (Equation (4)):

(4) $$\mathrm{Specificity}=\frac{\mathrm{tn}}{\mathrm{tn}+\mathrm{fp}}=1-\alpha ,$$

Ideally, an assay will have high sensitivity and specificity, with low false-negative and false-positive rates, meaning the assay allows to make decisions that are close to those made using the gold reference method. If the assay were perfect, the sensitivity and specificity would be 100%, and the false-negative rate and false-positive rates would be 0%. However, usually, an increase in sensitivity will imply a decrease in specificity, and vice versa, as they are inversely proportional [29].

### 2.4. Development of an ASSURED-Designed Sampling Preparation Kit

To overcome the challenges outlined in the introduction, a new sample preparation kit was developed and tested. A user-centered design was employed to ensure a simple and intuitive kit that can be used by untrained personnel. The purpose of user-centered design is to create a product that will be used in practice as it is intended to be used, requiring minimum effort by the end user [30]. Applying a user-centered design framework ensures that the sample preparation kit also complies with the ASSURED framework, particularly ensuring the kit is user friendly.

A modified user-centered design process was followed, as outlined by Kangas and Kinnunen [31], namely: (1) technological research; (2) initial feature requirements; (3) prototype testing; (4) pilot implementation; and (5) pilot field test.

**Figure 1:**
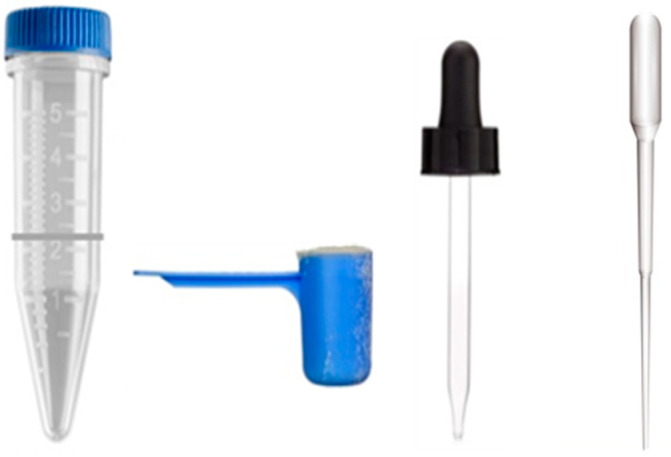
Sample preparation kit. From left to right, a sample tube with a marker line for 10 mL, a ½ tablespoon scoop, and an eyedropper or Pasteur pipette for sample deposition.

(1) Technological research. The initial technological research was conducted by Waller et al. [17], in which it was identified that a user-friendly, affordable sample preparation kit was needed to comply with the ASSURED criteria of the assay.

(2) Initial feature requirements. Initial feature requirements were identified by the three key action components of the sample preparation: sample deposition, sample weight, and sample dilution.

(3) Prototype testing. In this case, a simple and inexpensive eyedropper, glass Pasteur pipette, or plastic Pasteur pipette were tested to replace the micropipette (i.e., to deposit the sample on the paper); a ½ tablespoon scoop was tested to replace the analytical balance (i.e., to weigh the food sample); and a conical tube marked with a line that indicates a specific volume was tested to replace the volumetric pipette (i.e., for sample dilution) (Figure 1). Vortexing the sample was replaced by vigorously shaking for 10 s. Each tool in the sample preparation kit was tested for its internal analytical error by weighing water (eyedropper, Pasteur pipettes, and conical tube) and corn flour (sample scoop) on an analytical balance with five replications to estimate the expected increase in random error using the sample preparation kit compared to the analytical laboratory tools.

(4) Pilot implementation. The kit’s total CV_w_% variability was compared to the original method’s total CV_w_% (15.9%) by measuring one Mexican corn flour sample × 16 replicates to assess variability in precision (i.e., the closeness between multiple measurements).

(5) Pilot field test. Commercial samples collected in Mexico were analyzed using the sample preparation kit and the paper-based assay and compared to its reference values (obtained by AES), and the values obtained using the more precise laboratory tools. For iron determination, 1 scoop (2.5 g) was used to take an amount of sample and placed it into the volumetric test tube marked at the 40 mL line. Then, acidified solution (0.25 M HCl) was added until reaching the 40 mL mark. Samples were shaken for 10 s and let settle for 30 min. An aliquot of the supernatant was taken with a Pasteur pipette, and a drop was deposited on the paper-based sensor. The color was let develop for 5 min and then measured using a smartphone with the Nu3Px app as described before [17].

### 2.5. Statistical Analysis

Data were statistically analyzed using IBM SPSS 24 [32] and Microsoft Excel, including means, standard deviations, confidence intervals, % coefficient of variation (CV%), Pearson coefficient (r), determination coefficient (R^2^), bivariate correlations, total analytical error, paired sample *t*-test, Levene’s test for homogeneity of variances (F-test), McNemar test, and chi-square test (χ^2^). The degree of agreement was analyzed using bivariate correlations (*p* < 0.05) on method comparison plots. Bland–Altman plots were constructed, in which the reference method (AES) was plotted on the *x*-axis, and the difference between the novel method (paper-based) and the AES was plotted on the *y*-axis. The majority of data points should be within 1σ (68%), with acceptable methods having almost all data points within 1.96σ (95%). The σ of the Bland–Altman plots are known as upper and lower limits, which is the σ of the differences, plotted +/− from the bias (mean of the differences) [33]. Data points outside 1.96σ are not ideal but may be considered outliers if proven to be the case.

## 3. Results

**Figure 2:**
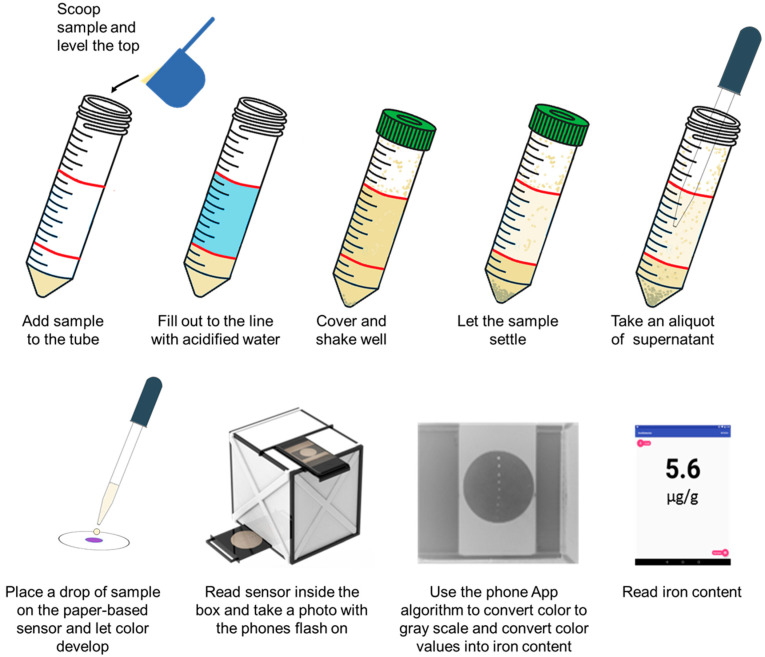
Schematic indicating the sample collection, preparation, and deposition on the paper-based sensor using simple tools as well as its detection and readout as shown previously [17].

A schematic of the sample preparation and readout is presented in Figure 2. This figure shows the sample collection and steps before adding a drop of sample on the paper-based sensor. The sample is then read and measured using the smartphone and the app as described before [17].

### 3.1. Characterization of Mexican Corn Flours

Table 1 characterizes the mineral contents of nixtamalized corn flours collected at various markets in Mexico. For completeness, a total of 13 minerals were analyzed. All samples were collected in Querétaro, except for sample #5 (Cuetzalan, Mexico) and samples #7 and #18 (Saltillo, Mexico). Samples #19–29 were produced locally in Querétaro. Samples #1–18 were produced in other parts of the country.

The nixtamalization process used in the processing of these corn flours is very well understood and described in detail in other studies [34,35,36].

Samples #19–21 were collected over a 3 h period from the same batch of fortified corn flour. The CV% within batch in the iron content was 23%. Samples #22–27 were collected over a 6.5 h period from the same batch, and the CV% of iron content was 38.8%. These CV%s are higher than the previously reported variability of iron-fortified corn flour of 15% [37] and highlight the variability of the industrial fortification mixing process within batch.

The company with the largest number of samples collected is company B (*n* = 14 samples). Of the 14 samples, the CV% of iron is 31.0%. This high variability emphasizes the randomness of the fortification process within company.

Under the Norma Official Mexicana (Mexico’s food standards) NOM-247-SSA1-2008, all corn flours marketed and distributed for consumption should be fortified with a minimum of 40 μg Fe/g flour (as ferrous sulfate or fumarate) [26]. Under this standard, there is no established maximum or upper limit for iron content in fortified flours. Based on the AES and paper-based sensor results, all but 2 (#17 and #22) of the 29 flours met this minimum requirement.

### 3.2. Replication Experiment

A between-day replication experiment was conducted over 2 weeks as suggested by Westgard [24]. Results can be found in Table 2. The average estimated random error was calculated to be 12% variation.

### 3.3. Comparison of Methods Experiment

**Figure 3:**
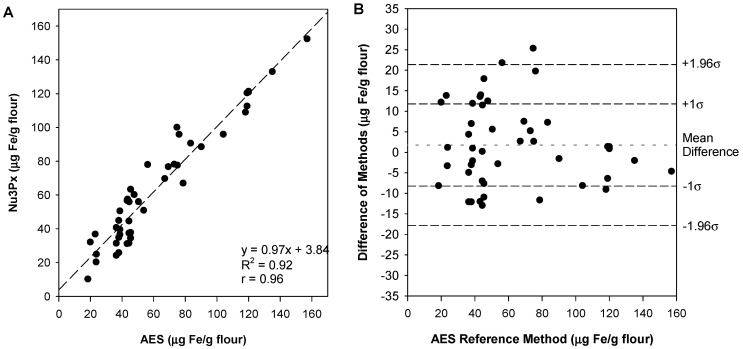
Comparison of methods plot (**A**) and Bland-Altman plot (**B**). The comparison of methods plot depicts bias, with the linear regression line quantifying systematic error. A linear regression line was fit (y = 0.97x + 3.84; R^2^ = 0.92). The Bland–Altman plot depicts variance. An acceptable variance will have 68% of data points within 1σ, and almost all (95%) within 1.96σ. Each σ indicates the standard deviation of differences.

A minimum of 40 samples is necessary to conduct the comparison of methods experiment. These were collected from local markets (*n* = 25) and fortified in laboratory (*n* = 20) and analyzed using both reference and new methods of measurement. The comparison plot (Figure 3) shows the error and variation associated with both measurements. If the two methods were identical, the linear regression line would have a slope of 1 and a y-intercept of 0. When sources of systematic error are present, the linear regression line is used to determine systematic error via the slope’s digression from 1 (proportional error) and the y-intercept’s digression from 0 (constant error).

The current sampling procedure uses 2.5 g of flour and 10 mL of acid. However, this sampling procedure was designed to not exceed a maximum iron concentration of 115 μg Fe/g flour. Seven of the samples have iron concentrations higher than 115 μg Fe/g flour. Following the recommended sampling preparation procedure as is, the methods comparison plot demonstrated large systematic error (y = 0.8085x + 11.835, R^2^ = 0.83). Because the R^2^ value of 0.83 is under 0.99, a better determination of systematic error is via the determination of bias (mean of differences). The bias (mean ± SD) was calculated to be −0.12 ± 14.1 μg Fe/g flour.

Due to the low R^2^ value, it was apparent that overestimation at higher concentrations (115 μg Fe/g flour) due to the color saturation of the assay was skewing the linear regression line. Thus, in response, the samples over 115 μg Fe/g flour were diluted using a larger volume (i.e., 25 mL dilute acid instead of 10 mL) and reanalyzed using this dilution factor to modify the output. By doing so, the overall linear regression equation (y = 0.97x + 3.84; R^2^ = 0.92) and the average bias (1.79 ± 9.99 μg Fe/g flour) indicated less systematic error. This demonstrates that dilution for higher concentration samples is a feasible modification to maintain a lower systematic error (Figure 3A).

The comparison of methods data can be transformed to fit a Bland–Altman plot (Figure 3B), which displays the variability of the method by plotting the reference method on the x-axis and the difference between the two methods on the y-axis. In addition, plotted are ±1.96σ and ±1σ of the differences (σ = 9.99 μg Fe/g flour). The majority of the data points (68%) should fit within ±1σ, and almost all of the data points (95%) should lie within ±1.96σ [33].

A paired sample *t*-test was conducted to understand the similarity between the two methods’ true mean values. The null hypothesis was that the true mean difference between the methods is equal to 0. Based on the data, we failed to reject the null hypothesis (*p* > 0.05). An F-test using Levene’s test was used to evaluate the homogeneity of variance. The null hypothesis was that the variances were similar. We failed to reject the null hypothesis (*p* > 0.05).

A contingency table (Table 3) was constructed comparing the method comparison data to Mexico’s fortification policy (<40 μg Fe/g flour, reject; ≥40 μg Fe/g flour, pass). From this table, qualitative performance parameters false-positive rate (21.4%), false-negative rate (16.1%), sensitivity (83.9%, CI_95%_: 70.9–96.8%), and specificity (78.6%, CI_95%_: 57.1–100.0%) were calculated.

When validating quantitative methods that determine qualitative decisions, the AOAC International recommends chi-square and McNemar tests to assess differences between methods [38]. A chi-square test was performed to compare the classifications of the two methods. The null hypothesis was that Nu3Px and AES made classifications independent from each other. We rejected the null hypothesis that the two classifications are independent of one another (Pearson χ^2^ = 16.411, *p* < 0.01). For the McNemar test, the null hypothesis was that the acceptance and rejection percentages are equal between the two methods. We failed to reject the null hypothesis and conclude that the two proportions were not statistically different, *p* = 0.727 (two-sided).

### 3.4. Development of a Sample Preparation Kit

Prototype testing. The sample preparation kit consisted of a simple tool to deposit the sample (eyedropper, plastic Pasteur pipette, or glass Pasteur pipette), a tube for sample dilution, and ½ tablespoon scoop for sample weight. The variability of the sample preparation kit tools was determined by measuring water or flour, weighing the amount measured, and calculating the CV% of several replicates (*n* = 5). The eyedropper was found to have a CV% of 7.24%, the plastic Pasteur pipette was found to have a CV% of 2.75%, and the glass Pasteur pipette was found to have a CV% of 6.48%, indicating a plastic Pasteur pipette as the most reliable tool for sample deposition. For sample dilution, the conical tube was found to have a CV% of 1.03%. For sample weight, the scoop was found to deliver a mean weight of 2.55 g and CV% of 3.02% (Table 4). In total, the use of the ASSURED sample preparation kit is expected to increase random error as compared to using the laboratory analytical tools, which inherently possess less random error due to their design (microliter pipette 0.0% CV, volumetric pipette 0.550% CV, and analytical balance 0.006% CV).

Pilot implementation. The initial precision of the sample prep kit was determined by measuring one Mexican corn flour sample (*n* = 16 replicates) and calculating its mean, % difference from the true mean, standard deviation, and CV%. Using the sample prep kit (with eyedropper) and the smartphone app, the mean of the sample was found to be 52.52 μg Fe/g corn flour. The AES true value was 50.4 μg Fe/g corn flour, for a % mean difference of 4.12%. Between the 16 replicates, the standard deviation was 7.44 μg Fe/g corn flour, with a CV_w_% of 14.17%. These findings warranted further exploration of the precision of the sample prep kit.

Pilot field testing. On average, the plastic Pasteur pipette deposited 33.34 ± 0.92 μL of supernatant (*n* = 5 replicates) or approximately 6.7 times the amount of supernatant that is deposited using a 5 μL conventional pipette. Because more iron is being deposited onto the detection zone, a stronger response was detected using the dilution procedure as is. Additional diluting volumes (i.e., 20, 40, and 70 mL) were tested; the final diluting volume of 40 mL showed the most accurate results. Therefore, the dilution procedure was modified to 2.5 g flour in 40 mL of 0.25 M HCl, and this dilution factor was applied to the output.

Twenty-five commercial samples were tested using the sample prep kit (plastic Pasteur pipette, conical tube with a line to 40 mL, and ½ tablespoon scoop) and a dilution modification as indicated before (Figure 1). The results using the sample kit significantly correlated to the output using the laboratory precise tools (bivariate correlation r = 0.914, *p* < 0.01) as well as the AES reference output (bivariate correlation r = 0.952, *p* < 0.01). After a paired *t*-test, we failed to reject the null hypothesis that the true mean differences between the laboratory precise tools and the sample prep kit are different from 0 (*p* > 0.05).

## 4. Discussion

The purpose of this study was to validate (i.e., quantify total error) a paper-based, smartphone-assisted assay for the determination of iron in fortified flours, also known as Nu3Px [17], using commercial fortified nixtamalized corn flour samples collected from several companies and collection points in Mexico. Additionally, these samples’ mineral profiles were characterized and can be compared to the Mexican food standards (NOM) compliance [26]. A sample prep kit that aligns with the WHO’s ASSURED guidelines was developed and pilot tested by comparing its error performance parameters to that of Nu3Px using conventional laboratory tools. It was found that Nu3Px performed within acceptable error parameters: 12% random error and 1.79 ± 9.99 μg Fe/g flour systematic error. Using both the gold standard method of analysis and Nu3Px results in a similar classification of samples under Mexican regulations. Though most of the corn flours collected complied with current regulations, these samples failed to comply with theoretical fortification parameters recommended by experts for upper limits.

While new methods for clinical diagnostics have published acceptable error ranges (i.e., blood iron, TE < 20% [24]), conventional food matrices (i.e., not food formulated for specific medical needs such as infant formula) often do not have well documented acceptable error ranges. This is largely due to the different effects of misdiagnosis. For example, while false-positive or -negative results from an assay responsible for determining a clinical diagnosis (i.e., HIV or pregnancy) directly affect people’s lives, product adulteration and misbranding, in the case that the food has less nutrient addition than specified by the law, will result in economic impacts on the food company, which may include product seizures, imprisonment, or fines [39]. The economic impacts of food piracy have been estimated to account for USD $200 billion in the industry [40].

Allen et al. argue that fortification policy enforcement (quality control) relies on accurate, precise, and reproducible methodologies [37]. Their recommendations state that monitoring technologies should be able to measure the micronutrient content such that it is known whether a sample meets the target fortification level (TFL) and is within the minimum fortification level (minFL) and the maximum fortification level (maxFL). In the case of iron, minFL and maxFL are equivalent to the legal minimum level (LminL) and the maximum tolerable level (maxTL), respectively. The LminL and maxTL are used for policy enforcement and any applied retribution (i.e., fines if the product is found to have iron content outside of the limits) for not meeting compliance, specific to each country’s policies. Equations to estimate LminL and maxTL (μg Fe/g flour) are shown below (Equations (5) and (6)):

LminL = minFL = TFL × (1 − (2 × Fe CV% ÷ 100))(5)

maxTL = maxFL = TFL × (1 + (2 × Fe CV% ÷ 100))(6)

For iron fortification in nixtamalized corn flour, the Fe CV% is 15% [37]. Based on the Mexican policy guidelines [26], the TFL is 40 mg Fe/kg. Then, the calculated LminL and maxTL based on Mexico’s known TFL is 28 and 52 μg Fe/g flour, respectively. Thus, it is necessary that the validated paper assay will have a limit of detection below the LminL (28 μg Fe/g flour) and a maximum in the working range above the maxTL (52 μg Fe/g flour) for it to be effectively used to assess compliance. Furthermore, bias (systematic error; mean of the differences between reference and novel methods) should be kept to under 6 μg Fe/g flour (i.e., 25% of the range from LminL to maxTL) for each parameter to be distinguishable from the others. The bias (systematic error, mean ± standard deviation) of the paper-based sensor is 1.79 ± 9.99 μg Fe/g flour, which complies with the allowable mean systematic error.

Random error. Regarding allowable random error for CV_b_%, performance targets to meet can be determined by comparing CV% performance of similar paper-based assays in the literature. Mentele et al. measured iron in aerosols on a paper-based assay and a computer scanner with a CV% = 26.1% [21]. Martinez et al. measured glucose and protein in urine on a paper-based assay with a camera phone with CV% between 15.5–21.7% and 16.6–29.2%, respectively [20]. Thus, a reasonable performance target to meet is CV_b_ ≤ 25%, which is an improvement to other paper-based assays, such as that of Mentele et al. [21]. Consequently, the CV_b_% of 12.0% complies with the allowable random error.

Total error. The final performance parameters are demonstrated in Table 5, with a 12% random error and a bias (systematic error; difference of means) of 1.79 ± 9.99 μg Fe/g flour (Table 5).

Compliance with current and theoretical regulations. Table 6 shows the paired results for both AES and Nu3Px for each sample and classifies each sample whether it was within or outside of the theoretical policy’s allowable range according to Allen et al. (28 to 52 μg Fe/g flour) and whether each sample was within or outside of Mexico’s policy allowable range (≥40 μg Fe/g flour) [37]. Based on Mexico’s current policy [26], Nu3Px agreed 100% of the time with the classification of Mexican samples (*n* = 25) based on AES results. However, according to Allen et al.’s theoretical policy limits using calculated LminL and maxTL, Nu3Px would have provided false positives 24% of the time [37].

As Mexico’s policy currently stands, Nu3Px is a ready and applicable monitoring and evaluation tool for compliance, as 100% of the time, Nu3Px agreed with the gold reference method whether to reject the batch or not. However, if Mexico modifies its policy in the future to align with fortification policy-making experts, further research will need to be conducted to reduce the prevalence of false positives and negatives in the paper-based assay.

Other Central American countries that have corn flour fortification policies have adopted similar policies to Mexico’s, with only minimum fortification levels and no upper limits. Costa Rica’s policy requires a minimum of 22 mg Fe/kg flour [41], El Salvador’s policy requires a minimum of 40 mg Fe/kg flour [42], and Guatemala’s minimum requirement is 17 mg Fe/kg flour [41]. Thus, Nu3Px is expected to perform well under other Central American regulatory policies as well.

Qualitative performance parameters of Nu3Px. When quantitative assays are used to make qualitative decisions (i.e., binary decisions such as yes/no or reject/pass), several performance parameters are recommended to be calculated, such as the false-positive rate, false-negative rate, and sensitivity [27,38]. When comparing all 45 samples to Mexico’s current fortification policy as written in the NOM, Nu3Px showed a false-positive rate of 21.4%, a false-negative rate of 16.1%, sensitivity of 83.9%, and specificity of 78.6%. The samples that were closer to the cut-off point (40 μg Fe/g flour) were more likely to show disagreement with the AES due to the paper-based method’s inherent random error.

While there are no published allowances of specificity and sensitivity for new paper-based assays for micronutrients, we can compare Nu3Px’s performance to other published rapid, qualitative decision-making tools. iCheck Iodine, a rapid field test for checking iodine content in salt, reported a sensitivity of 92.4% and a specificity of 100% [43]. A similar detection method, the iCheck Iron, uses the bathophenantrolin reagent that reacts with reduced iron creating a deep magenta. The absorbance is then quantified with a photometer. This method has been validated using iron-fortified fish and soy sauces; however, evaluations of sensitivity and specificity were not included [44]. A paper-based assay for screening sickle cell anemia (yes/no presence) reported a sensitivity and specificity of 93% and 94%, respectively [45]. A microchip assay coupled with a smartphone to detect semen count above and below the WHO threshold (100,000 sperm/mL) was found to have a sensitivity of 92.86% and a specificity of 100% [46]. Though Nu3Px’s performance parameters are lower than these examples, it is important to note that none of the above-mentioned examples use messy food samples, a smartphone, simple tools for samples collection, and a paper-based assay for detection, all of which provide great challenges to overcome. Though there is still work to be done in improving the performance of Nu3Px, Mexico’s fortification program can still benefit from using this rapid monitoring tool, as it is more precise than the iron spot test (i.e., iron and potassium thiocyanate reaction) [47], which is the current internal monitoring method.

Sample characterization. Based on AES, two of the samples (#17 and 22) were lower than the minimum level per Mexico’s regulation (40 μg Fe/g flour), and 22 of the 25 samples contained amounts of iron greater than the theoretical maxTL of 52 μg Fe/g flour [37]. Low iron concentrations threaten the program’s ability for positive nutritional impact. On the other hand, high iron concentrations can have negative consequences for human health. It is known that when consumed in toxic quantities, iron accumulates in organs such as the liver, spleen, or kidneys [48]. For these reasons, Allen et al. recommend the addition of upper levels to fortification programs [37]. As demonstrated, Nu3Px can serve as an internal quality control checkpoint for food processors to monitor the levels of iron in the fortified flour, with a particular focus on ensuring toxic upper levels of iron are not met, as was the case in 72% of the samples collected, with iron levels reaching 3 times the target amount.

Sample preparation kit. The sample prep kit was piloted with measuring tools commonly available and affordable around the world. Plastic pipettes or eye droppers can be found at most pharmacies, ½ tablespoon scoop used in home cooking can be found in most kitchenware stores or simply manufactured, and a conical tube with a line drawn to 10 or 40 mL, depending on the diluting volume to be used, is an inexpensive alternative that can easily be manufactured.

In the case of the sample prep kit, the bias (systematic error, mean of the differences) was +2.12 μg Fe/g flour, or a % mean difference of 4.12%. The results from measuring commercial samples in Mexico were comparably similar to the Nu3Px output using laboratory precise tools. This meets the performance requirements to be used in the Mexican corn flour fortification program.

There are several limitations associated with the method validation experiment and the development of a sample preparation kit. First, the method validation experiment conducted herein obliges by the minimum sample requirement (*n* = 40); however, a larger sample size is always preferred. The method has not been validated across individuals or laboratories, i.e., an inter-laboratory validation. This is the preferred form of method validation, though an expensive process. In cases where expenses are limited, intra-laboratory method validation is suggested [49]. Nu3Px showed a 24% false-positive rate when comparing the theoretical policy limits. If countries such as Mexico are to modify their policies to the recommended policy limits, further research is warranted to reduce the prevalence of false-positive to implement Nu3Px.

Due to the method’s logarithmic calibration curve, at high concentrations (i.e., above 115 μg Fe/g flour), the results are variable. This can pose an issue for an end-user food company if their flours tend to fall to the higher concentration values, as is the case for Mexico. Sample dilution is the best strategy, though it has to be corrected in the final calculation, as was demonstrated.

The sample preparation kit’s design assumes that the end user has access to an eyedropper, a conical tube with a volumetric marking, and a tablespoon scoop. Secondly, the error quantification is specific to the tools used and is presented as an example case study. These experiments would need to be repeated upon finalizing the sample preparation kit design and manufacturing.

## 5. Conclusions

A sample preparation kit was developed that aligns with the WHO’s ASSURED criteria (i.e., Affordable, User friendly, and Equipment-free), which has similar precision to using analytical methods but at a lower cost and greater access. The sample preparation kit, coupled with the smartphone app and paper-based assay, measures iron within the performance parameters required for the application to corn flour fortification programs, such as the case in Mexico. A validated ASSURED-designed technology can be useful for monitoring fortified staple foods, specifically ensuring that the flours meet government specifications.

## Figures and Tables

**Table 1:** Elemental characterization of commercial nixtamalized corn flour samples collected in Mexico.

Company	Sample ID	N (%)	P (%)	Mg (%)	K (%)	Ca (%)	S (%)	B (ppm)	Mn (ppm)	Cu (ppm)	Zn (ppm)	Al (ppm)	Na (ppm)	Fe (ppm)
A	1	1.27	0.242	0.081	0.30	0.21	0.089	1.2	4.6	1.0	55.1	2.7	113	53.7
	2	1.38	0.245	0.089	0.30	0.21	0.100	1.2	4.0	1.1	51.0	4.4	79.5	50.4
B	3	1.25	0.265	0.091	0.31	0.08	0.091	1.2	4.9	1.0	50.1	14.8	89.4	78.6
	4	1.28	0.271	0.094	0.32	0.06	0.090	1.0	4.4	0.8	57.7	1.3	20.4	83.3
	5	1.35	0.303	0.098	0.32	0.08	0.100	0.9	4.5	0.8	52.7	1.6	15.1	75.0
	6	1.40	0.266	0.095	0.32	0.08	0.091	1.0	4.5	0.9	68.4	5.1	44.0	119.0
	7	1.22	0.272	0.101	0.31	0.07	0.09	0.9	4.7	1.1	79.1	3.5	48.4	120.0
	8	1.19	0.286	0.104	0.34	0.07	0.094	1.3	4.8	1.0	89.0	1.2	42.2	157.0
	9	1.34	0.276	0.091	0.32	0.08	0.091	1.3	4.7	1.1	47.9	5.0	98.1	76.1
	10	1.32	0.299	0.105	0.33	0.09	0.097	1.1	4.8	1.1	76.0	3.0	32.1	118.0
	11	1.25	0.256	0.092	0.30	0.06	0.087	1.0	3.8	1.0	56.8	3.3	42.5	90.1
	12	1.27	0.267	0.092	0.31	0.07	0.092	1.4	4.3	1.0	43.7	3.9	98.1	67.0
	13	1.33	0.273	0.096	0.31	0.06	0.086	1.0	4.5	0.9	64.1	1.0	39.1	104.0
	14	1.26	0.249	0.085	0.28	0.08	0.086	1.3	4.2	1.0	30.9	3.4	80.6	43.5
	15	1.27	0.245	0.088	0.29	0.06	0.084	1.0	3.8	0.9	79.4	1.7	29.7	135.0
	16	1.26	0.249	0.091	0.29	0.06	0.087	1.0	4.0	0.9	72.5	3.7	44.2	119.0
C	17	1.25	0.251	0.087	0.30	0.13	0.088	1.5	3.2	0.6	20.0	1.5	30.0	19.9
D	18	1.29	0.266	0.101	0.31	0.06	0.091	1.1	4.5	1.0	69.2	2.9	55.9	114.0
E	19	1.20	0.265	0.096	0.36	0.28	0.083	1.4	4.5	0.8	53.2	6.9	66.8	47.9
	20	1.20	0.281	0.099	0.35	0.34	0.086	1.4	4.8	1.2	54.7	8.0	41.6	68.7
	21	1.24	0.288	0.095	0.35	0.37	0.085	1.5	4.6	0.7	55.0	7.6	48.1	77.2
	22	1.24	0.249	0.090	0.33	0.26	0.082	1.2	4.2	0.6	15.5	4.8	60.1	18.4
	23	1.28	0.262	0.093	0.34	0.29	0.091	1.6	4.6	1.0	49.5	9.1	61.7	73.0
	24	1.24	0.273	0.098	0.35	0.30	0.087	1.3	4.7	0.9	58.2	6.6	54.5	74.7
	25	1.28	0.255	0.091	0.35	0.31	0.094	1.5	4.3	0.8	47.5	7.3	49.8	56.2
	26	1.23	0.238	0.087	0.33	0.32	0.089	1.3	4.2	0.8	44.8	4.4	45.0	44.5
	27	1.24	0.287	0.101	0.35	0.30	0.089	1.5	4.8	0.8	108	4.3	36.6	69.2
F	28	1.32	0.345	0.097	0.32	0.14	0.095	1.7	7.0	0.8	44.9	3.1	13.1	47.7
	29	1.29	0.328	0.094	0.31	0.14	0.092	1.5	7.0	0.9	41.9	2.8	13.7	48.3

**Table 2:** Replication between-day experiment. The total expected amount of random error within the method was determined, and CV%s are shown.

*n* Days						
	3	3	2	2	2	Average RE
CV%	11%	19%	14%	4%	11%	12%

**Table 3:** Contingency table describing pass/reject measurements (*n* = 45) using AES and Nu3Px based on Mexico’s current fortification policy.

		AES Classification		Total
		Pass ^1^	Reject ^2^	
Nu3Px Classification	Pass	26	3	29
	Reject	5	11	16
	Total	31	14	45

^1^ Pass if iron measurement is (≥40 μg Fe/g flour). ^2^ Reject if iron measurement is (<40 μg Fe/g flour).

**Table 4:** Comparison of random error between sample prep kit and laboratory tools.

Step in Sample Preparation	Matrix Tested	Sample Kit Tool	CV% (*n* = 5)	Laboratory Precision Tool	CV% (*n* = 5)
Deposition	Water	Eyedropper	7.24	Microliter Pipette	0
Deposition	Water	Plastic pipette	2.75	-	-
Deposition	Water	Glass pipette	6.48	-	-
Dilution	Water	Conical tube	1.03	Volumetric Pipette	0.55

**Table 5:** Error quantification. Random and systematic errors are quantified at the preliminary and final stages of method validation.

Type of Analytical Error	Preliminary Error Evaluation	Final Error Evaluation
Random Error	15.9%	12.0%
Systematic Error (Constant)	1.01 μg Fe/g flour	1.79 ± 9.99 μg Fe/g flour
Systematic Error (Proportional)	13.1%	

**Table 6:** Classification of corn flour collected in Mexico (*n* = 25) based on iron determinations using AES and Nu3Px based on Mexico’s policy ^1^ and Allen’s theoretical estimation of parameters for fortification policy ^2^.

ID	AES (μg/g Flour)	AES (Theoretical Policy)	AES (Actual Policy)	Nu3px (μg/g Flour)	Nu3Px (Theoretical Policy)	Nu3Px (Actual Policy)	Sensitivity Based on Theoretical Limits	Sensitivity Based on Actual Policy
1A	53.7	High	Good	50.9	Good	Good	No match	Match
2A	50.4	Good	Good	56.2	High	Good	No match	Match
3B	78.6	High	Good	66.9	High	Good	Match	Match
4B	83.3	High	Good	90.6	High	Good	Match	Match
5B	75	High	Good	77.7	High	Good	Match	Match
6B	119	High	Good	120.4	High	Good	Match	Match
7B	120	High	Good	120.9	High	Good	Match	Match
8B	157	High	Good	152.4	High	Good	Match	Match
9B	76.1	High	Good	95.9	High	Good	Match	Match
10B	118	High	Good	108.9	High	Good	Match	Match
11B	90.1	High	Good	88.6	High	Good	Match	Match
12B	67	High	Good	69.7	High	Good	Match	Match
13B	104	High	Good	95.9	High	Good	Match	Match
14B	43.5	Good	Good	57.5	High	Good	No match	Match
15B	135	High	Good	133.0	High	Good	Match	Match
16B	119	High	Good	112.6	High	Good	Match	Match
17C	19.9	Low	Low	32.1	Good	Low	No match	Match
18D	114	High	Good	89.9	High	Good	Match	Match
22E	18.4	Low	Low	10.3	Low	Low	Match	Match
23E	73	High	Good	78.2	High	Good	Match	Match
24E	74.7	High	Good	100.0	High	Good	Match	Match
25E	56.2	High	Good	78.0	High	Good	Match	Match
26E	44.5	Good	Good	55.9	High	Good	No match	Match
27E	69.2	High	Good	76.7	High	Good	Match	Match
28F	47.7	Good	Good	60.2	High	Good	No match	Match

^1^ Mexico NOM: iron content ≥ 40 µg/mL. ^2^ Allen’s theoretical estimation of parameters for fortification policy: ≥28 and ≤52 µg/mL.

## Data Availability

Despite all information has been provided herein, raw data are available upon request to the corresponding author.
